# S100 Calcium Binding Protein Family Members Associate With Poor Patient Outcome and Response to Proteasome Inhibition in Multiple Myeloma

**DOI:** 10.3389/fcell.2021.723016

**Published:** 2021-08-16

**Authors:** Minxia Liu, Yinyin Wang, Juho J. Miettinen, Romika Kumari, Muntasir Mamun Majumder, Ciara Tierney, Despina Bazou, Alun Parsons, Minna Suvela, Juha Lievonen, Raija Silvennoinen, Pekka Anttila, Paul Dowling, Peter O’Gorman, Jing Tang, Caroline A. Heckman

**Affiliations:** ^1^Institute for Molecular Medicine Finland – FIMM, HiLIFE – Helsinki Institute of Life Science, iCAN Digital Cancer Medicine Flagship, University of Helsinki, Helsinki, Finland; ^2^Research Program in Systems Oncology, Faculty of Medicine, University of Helsinki, Helsinki, Finland; ^3^Department of Hematology, Mater Misericordiae University Hospital, Dublin, Ireland; ^4^Department of Biology, National University of Ireland, Maynooth, Ireland; ^5^Department of Hematology, Helsinki University Hospital Comprehensive Cancer Center, University of Helsinki, Helsinki, Finland

**Keywords:** multiple myeloma, S100 protein family, drug resistance, proteasome inhibitors, panobinostat

## Abstract

Despite several new therapeutic options, multiple myeloma (MM) patients experience multiple relapses and inevitably become refractory to treatment. Insights into drug resistance mechanisms may lead to the development of novel treatment strategies. The S100 family is comprised of 21 calcium binding protein members with 17 S100 genes located in the 1q21 region, which is commonly amplified in MM. Dysregulated expression of S100 family members is associated with tumor initiation, progression and inflammation. However, the relationship between the S100 family and MM pathogenesis and drug response is unknown. In this study, the roles of S100 members were systematically studied at the copy number, transcriptional and protein level with patients’ survival and drug response. Copy number analysis revealed a predominant pattern of gains occurring in S100 genes clustering in the 1q21 locus. In general, gains of genes encoding S100 family members associated with worse patient survival. However, S100 gene copy number and S100 gene expression did not necessarily correlate, and high expression of *S100A4* associated with poor patient survival. Furthermore, integrated analysis of S100 gene expression and *ex vivo* drug sensitivity data showed significant negative correlation between expression of S100 family members (*S100A8*, *S100A9*, and *S100A12*) and sensitivity to some drugs used in current MM treatment, including proteasome inhibitors (bortezomib, carfilzomib, and ixazomib) and histone deacetylase inhibitor panobinostat. Combined proteomic and pharmacological data exhibited significant negative association of S100 members (S100A4, S100A8, and S100A9) with proteasome inhibitors and panobinostat. Clinically, the higher expression of *S100A4* and *S100A10* were significantly linked to shorter progression free survival in patients receiving carfilzomib-based therapy. The results indicate an association and highlight the potential functional importance of S100 members on chromosome 1q21 in the development of MM and resistance to established myeloma drugs, including proteasome inhibitors.

## Introduction

Multiple myeloma (MM) is the second most common hematologic malignancy characterized by the expansion of clonal plasma cells. The advent of novel agents, such as immunomodulatory drugs (IMiDs) and proteasome inhibitors (PIs), have markedly improved patient outcome (Fonseca et al., 2017). Although the majority of patients initially respond to therapy, eventually patients experience multiple relapses, and ultimately become refractory to treatment (Rajkumar, 2011). It is therefore important to identify factors predicting response to treatment, decipher drug resistance mechanisms, and guide the development of future treatment approaches.

Previous studies have demonstrated cytogenetic abnormalities can be used to determine MM patient prognosis (Palumbo et al., 2015; Manier et al., 2017). The gain/amplification of chromosome 1q21 (1q21+) is among the most frequent chromosomal aberrations in MM, occurring in approximately 40% of patients with greater prevalence in relapsed patients than those with newly diagnosed disease (Marzin et al., 2006). Several studies have shown that 1q21+ associates with poor overall survival in MM patients, independent of other high-risk chromosomal abnormalities (Hanamura et al., 2006; Giri et al., 2020). In addition, 1q21+ confers inferior outcome in patients treated with IMiDs and PIs (Shaughnessy, 2005; Nahi et al., 2016; Schmidt et al., 2019). Investigations of the genes within the 1q21 region have provided some insight into the mechanisms that could contribute to the poor outcome of patients with this aberration. These include *CKS1B* (Shaughnessy, 2005), *MCL1* (Samo et al., 2018), *IL6R* (Teoh et al., 2020), and *ADAR* (Teoh et al., 2020), all of which have been shown to promote MM progression. Inhibitors of the Mcl-1 protein have shown potent anti-myeloma activity in 1q21-amplified MM patients (Slomp et al., 2019). However, the influence of other 1q21 genes on MM pathogenesis and patient response to treatment is largely unknown.

The S100 protein family is the largest subgroup of calcium binding EF-hand type proteins and consists of at least 21 members that tend to form homodimeric and/or heterodimeric complexes with each other (Heizmann et al., 2002). The majority (*S100A1*-*S100A16*, *TCHLL1*) are in the 1q21 region, while *S100B*, *S100G*, *S100P*, and *S100Z* are located at 21q22, Xp22, 4p16, and 5q14, respectively. All S100 members except for S100G are Ca^2+^ sensor proteins and participate in multiple biological processes, including cell proliferation, growth, motility, apoptosis, differentiation, inflammatory response, Ca^2+^ homeostasis, protein synthesis, transcription, and membrane permeability (Donato et al., 2013; Kuberappa et al., 2016). However, S100 proteins showed cell-specific expression patterns and non-redundant functions by interacting with a unique set of target proteins (Donato, 2001). Certain S100 proteins have intercellular and/or extracellular functions. Within cells, S100 proteins interact with multiple target proteins and exert a broad range of cellular functions, including cell proliferation, differentiation, calcium homeostasis and energy metabolism. Extracellular S100 proteins induce proinflammatory responses and immune escape by activating JAK-STAT, NF-κB, and MAPK pathways by binding to toll-like receptor 4 (TLR4), receptor for advanced glycation end products (RAGE), and other cell surface receptors (Donato et al., 2013). Dysregulated expression of many S100 family members have been described in various types of cancers and implicated in tumor cell proliferation, invasion, metastasis, angiogenesis, and immune evasion (Bresnick et al., 2015). *S100A4* has been shown to promote acute myeloid leukemia (AML) survival (Alanazi et al., 2020) and *S100A8* is considered as a prognostic marker for AML (Nicolas et al., 2011). Upregulation of *S100A6* correlates with poor outcome of pancreatic cancer patients (Vimalachandran et al., 2005). Overexpression of *S100A8* and *S100A9* associates with the progression of many solid tumors, including prostate (Grebhardt et al., 2014), colorectal (Ichikawa et al., 2011), and breast cancer (Goh et al., 2017). *S100A13* has been shown to promote melanoma metastasis and chemoresistance (Azimi et al., 2014). Furthermore, several studies have reported some S100 members participated in drug resistance: higher expression of *S100A8* and *S100A9* leads to glucocorticoid resistance in *MLL* rearranged infant acute lymphoid leukemia (Spijkers-Hagelstein et al., 2012), while our previous study revealed elevated expression of *S100A8* and *S100A9* negatively associate with sensitivity to the BCL2 inhibitor venetoclax in AML (Karjalainen et al., 2019). Some S100 proteins (*S100A3*, *S100A9*, *S100A12*, and *S100A13*) were upregulated in normal plasma cells compared with MM cells (Zhan et al., 2002; Amit et al., 2021). In a recent study, *S100A4, S100A10*, and *S100A11* showed significant upregulation in MM non-responding *versus* responding patients treated with a combined regimen, including daratumumab, carfilzomib, lenalidomide, and dexamethasone (KARA-KRD), and their overexpression were also involved in clonal evolution of a primary refractory MM patient responding to KARA-KRD treatment (Cohen et al., 2021). Although S100 proteins are known to contribute to cancer progression and drug resistance, their functions in MM and possible effects on treatment response remain unclear.

The aims of this study are to investigate the roles of S100 in MM progression using cytogenetic profiling, copy number analysis, gene expression, protein expression, and clinical outcome data. Furthermore, by integrating gene expression, *ex vivo* drug response, and clinical response data, we aimed to determine whether S100 expression could guide treatment response, especially to established myeloma drugs, including proteasome inhibitors.

## Materials and Methods

### Patient Samples

Bone marrow (BM) aspirates and skin biopsies were obtained from 56 newly diagnosed and 115 relapsed/refractory patients after informed consent using protocols in accordance with the Declaration of Helsinki and approved by an ethical committee of the Helsinki University Hospital (study numbers 239/13/03/00/2010 and 303/13/03/01/2011). Mononuclear cells were isolated by Ficoll-Paque gradient centrifugation (GE Healthcare, Little Chalfont, Buckinghamshire, United Kingdom), followed by enrichment of CD138+ plasma cells using the EasySep^TM^ Human CD138 Positive Selection Kit (StemCell Technologies, Vancouver, BC, Canada). Cytogenetic data were generated using routine diagnostic fluorescence *in situ* hybridization technology following the European Myeloma Network 2012 guidelines (Caers et al., 2018). Fluorescence *in situ* hybridization (FISH) was performed at patient diagnosis using the following probes: Vysis IGH/FGFR3 (4p16/14q32, DC.DF), Vysis IGH/CCND1 (11q13/14q32, DC.DF), Vysis TP53 (17p13.1), Vysis 1q21 CKS1B/1p32 CDKN2C, and Vysis D13S319/13q34. Patient characteristics are described in Supplementary Table 1.

### RNA Sequencing and Analysis

RNA sequencing was performed for 116 MM patient samples as described previously (Kumar et al., 2017). Briefly, RNA was prepared from freshly isolated CD138+ cells with the Qiagen AllPrep kit (Qiagen, Hilden, Germany) and ribosomal RNA was depleted using the Ribo-Zero^TM^ rRNA Removal Kit (Epicenter, Madison, WI, United States), then reverse transcribed to double stranded cDNA (SuperScript^TM^ Double Stranded cDNA Synthesis Kit, Thermo Fisher Scientific, Carlsbad, CA, United States). Illumina-compatible RNA sequencing libraries were prepared using ScriptSeqTM technology and sequenced on IlluminaHiSeq^®^ 1500 or 2500 instruments (Illumina, San Diego, CA, United States). After pre-processing, filtered reads were aligned to the GRCh38 human reference genome using the STAR aligner tool (Dobin et al., 2013). Gene read counts were normalized using the reads per kilobase of transcript per million mapped reads (RPKM) method. A cutoff value >1 RPKM was used to filter the expressed *S100* genes. Data are available on the Center for Open Science platform OSF^1^.

### Exome Sequencing and Copy Number Analysis

Genomic DNA was isolated from CD138+ cells and skin biopsies (*n* = 168) with the DNeasy Blood & Tissue kit or AllPrep^®^ DNA/RNA/miRNA Universal kit (Qiagen). Exome capture was performed with The SeqCap^®^ EZ MedExome kit (Roche NimbleGen, Madison, WI, United States), SureSelect Clinical Research Exome kit, or SureSelect Human All Exon V5 kit (Agilent Technologies, Santa Clara, CA, United States) and exomes sequenced on a HiSeq^®^ 1500 or 2500 instrument. Gene copy numbers were estimated from exome sequencing data as previously described (Sulonen et al., 2011; Kumar et al., 2021). Copy number variants (CNVs) were identified using the CopyCat tool^2^. Patient with log2 copy number ratio ≥ 0.5 were considered as a gain while ratio <−0.6 were considered as deletion. Data are available in the OSF platform (see text footnote 1).

### Proteome Analysis

Proteome analysis was performed for 35 MM patient samples as described previously (Tierney et al., 2021). Briefly, CD138 + cells were lysed in RIPA buffer (Cell Signaling Technology, Danvers, MA, United States). The whole cell lysates were then digested and loaded onto a Q Exactive (Thermo Fisher Scientific, Hemel Hempstead, United Kingdom) high-resolution accurate mass spectrometer connected to a Dionex Ultimate 3000 (RSLCnano) chromatography system (Thermo Fisher Scientific, Hemel Hempstead, United Kingdom). Protein identification and quantification was performed using MaxQuant v1.5.2.8^3^. Perseus v.1.5.6.0^4^ was used for data analysis, processing, and visualization. Normalized label-free quantification intensity values were used as the quantitative measurement of protein abundance for subsequent analysis. The dataset is available on the OSF platform (see text footnote 1).

### Drug Sensitivity and Resistance Testing (DSRT) of MM Patient Cells

The sensitivity of CD138+ cells from the MM patient samples (*n* = 142) was assessed to up to 308 small molecule drugs including approved and investigational compounds as described previously (Majumder et al., 2017). The drugs were pre-plated onto 384-well plates in five concentrations in a 10,000-fold concentration range. The cells were suspended in RPMI 1640 medium supplemented with 10% fetal bovine serum, 2 mM L-glutamine, penicillin (100 U/ml), streptomycin (100 μg/ml), and 25% conditioned medium from the HS-5 human BM stromal cell line with 5000 cells added per well. After 72 h incubation at 37°C and 5% CO_2_, cell viability was measured using the CellTiter-Glo (CTG) assay (Promega, Madison, WI, United States) with a PHERAstar FS plate reader (BMG LABTECH, Ortenberg, Germany). A drug sensitivity score (DSS) was calculated based on a modified area under the dose-response curve calculation (Yadav et al., 2014). Selective drug sensitivity scores (sDSS) for the MM patient samples were calculated by subtracting the median DSS of BM mononuclear cells from eight healthy donors.

### Drug Testing for MM Cell Lines

KMS-12-BM, NCI-H929, U-266, SK-MM-1, OPM-2, and MM.1S cell lines were purchased from the American Type Culture Collection (ATCC, Wesel, Germany). All cell lines were cultured in RPMI-1640 medium, supplemented with 10–20% heat-inactivated fetal bovine serum (FBS), 2 mM L-glutamine, 100 U/mL penicillin, and 100 μg/ml streptomycin (Gibco, Thermo Fisher Scientific, Waltham, MA, United States) at 37°C in a humidified incubator with 5% CO2. The compounds were pre-plated onto 384-well plates in five concentrations in a 10,000-fold concentration range. The cells were seeded in 25 μl volume of medium at the density of 5000 cells/well. After 72 h, cell viability was measured using the CTG assay. The data were normalized to negative control (dimethyl sulfoxide vehicle only) and the positive controls wells (100 μmol/l benzethonium chloride).

### Data Validation

To validate our results, clinical, gene expression and genomic variant data (CNV) were obtained from the Multiple Myeloma Research Foundation (MMRF) Relating Clinical Outcomes in MM to Personal Assessment of Genetic Profile (CoMMpass) study (IA15a release)^5^^,^^6^. Specifically, the normalized gene expression table of Cufflinks gene FPKM and Exome per gene largest segment were used for gene expression and CNV analysis. Gene expression and CNV datasets include 892 and 1044 patient cases, respectively. A total of 54 paired MM samples were used to compare S100 gene expression between the diagnostic and the first relapse samples.

### Statistical Analysis

Statistical analyses were performed with R version 4.0.3 (2020-10-10) and Prism 9 (GraphPad, La Jolla, CA, United States). Drug response comparisons were computed with the Mann–Whitney *U* test and correlation analyses with the Spearman’s rank correlation coefficient test. Gene expression values were compared using the unpaired two-sample *t*-test. Baseline characteristics between patients with and without 1q21 gain or S100 gain were analyzed by the chi-square test and the Fisher’s exact test. Overall survival (OS) and progression-free survival (PFS) were calculated using the Kaplan-Meier method, and differences among survival curves were analyzed using a log-rank test. Cox proportional hazards regression analysis was used to examine the association of *S100* expression with OS, adjusting for potential confounders including age, gender, and cytogenetic alterations. All statistical tests applied in this study were two-tailed and *P*-values below 0.05 were considered significant.

## Results

### Prevalence and Prognostic Significance of *S100* Copy Number Change

**FIGURE 1:**
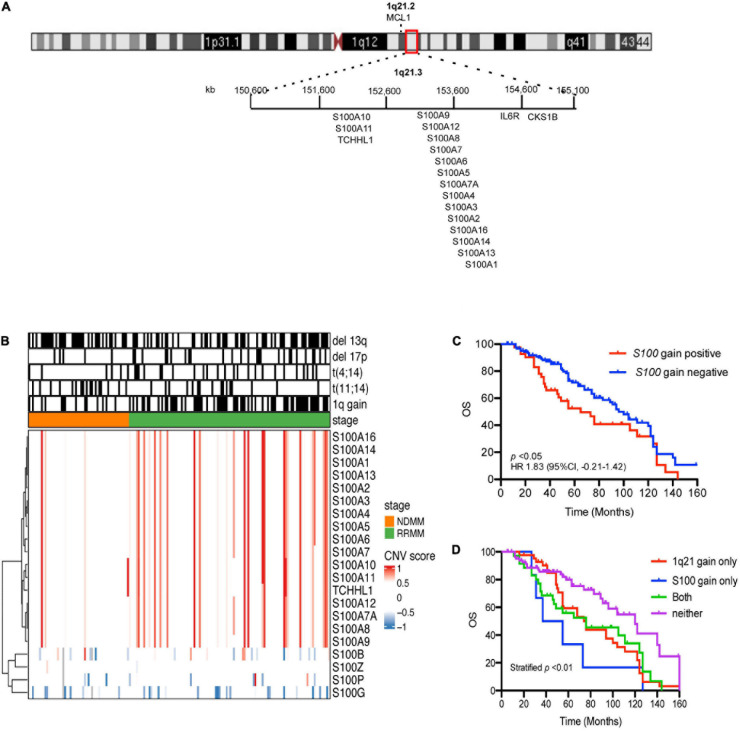
Prevalence and prognostic significance of *S100* copy number change. **(A)** Location of the 17 S100 genes on chromosome 1q21.3. **(B)** Hierarchical clustering of *S100* genes in CD138+ plasma cells from 168 MM patient samples, using Euclidean distance matrix and complete clustering method, based on CNV score. **(C)** The increased copy number of S100 genes correlated with poor overall survival. **(D)** Kaplan-Meier curves for MM patients with or without S100 gain. Because patients with S100 gain were also more likely to have 1q21 gain, four groups were used for OS analysis and included: neither 1q21 gain nor S100 gain (Neither), 1q21 gain present but no S100 gain (only 1q21 gain), S100 gain present but no 1q21 gain (only S100 gain), and 1q21 gain and S100 gain present (Both). CNV score > 0.5 indicated a gain event, while <–0.6 is a deletion event. CNV, copy number variation; FIMM, Institute for Molecular Medicine Finland; OS, overall survival; HR, hazard ratio; CI, confidence interval.

Cytogenetic data (Supplementary Table 1) showed 1q21 gain in 48.21% of samples (81/168), del(13q) 55.36% (93/168), del(17p) 19.64% (33/168), t(4:14) 19.04% (32/168), and t(11:14) 19.04% (32/168). Seventeen S100 genes (*S100A1, S100A2, S100A3, S100A4, S100A5, S100A6, S100A7, S100A7A, S100A8, S100A9, S100A10, S100A11, S100A12, S100A13, S100A14, S100A16*, and *TCHLL1*), located as a cluster on chromosome 1q21, exhibited copy number gains in 24.4% (41/168) of the MM samples (Figure 1A and Supplementary Table 2). Of the 168 MM patient samples analyzed, there were 4 newly diagnosed MM patients (NDMM) with S100 gain and 1q21 gain, no NDMM samples with S100 gain and no 1q21 gain, 10 NDMM samples with 1q21 gain and no S100 gain, 31 relapsed/refractory MM (RRMM) patients with S100 gain and 1q21 gain, 6 RRMM with S100 gain and no 1q21 gain, and 33 RRMM with 1q21 gain and no S100 gain (Figure 1B and Supplementary Table 2). Correlation analysis of S100 gain with disease stage and 1q21 gain showed S100 gains were detected more frequently in RRMM samples than samples from NDMM samples and co-occurred with 1q21 gain, indicating 1q21 gain is a major factor for S100 gain (Table 1). Copy number gains of *S100A9*, *S100A7*, *S100A7A*, and *S100A8* significantly correlated with poor overall survival in MM patients from our internal (FIMM) dataset (Supplementary Table 3). Overall, S100 genes in the 1q21 region show similar gains and have a negative impact on survival (Figure 1C). Since patients with S100 gain were more likely to have 1q21 gain, a 4-level measure was created for survival analysis. The median overall survival (OS) for each of the four categories was 120 months for no documented 1q21 or S100 gain, 76 months for 1q21 gain but no S100 gain, 46 months for S100 gain but no 1q21 gain, and 76 months for both S100 gain and 1q21 gain. Patients with 1q21 gain and without S100 gain and patients with S100 gain and without 1q21 gain were associated with significantly poorer outcome of in our FIMM dataset (Figure 1D). The CoMMpass dataset was used for validation and confirmed the high frequencies of S100 gene gains in 1044 samples (Supplementary Figures 1A,B). Integrated analysis of copy number variation (CNV) and gene expression showed that only *S100A13* had a significant positive correlation (*r* = 0.42) between copy number gains and gene expression levels in the FIMM dataset (Supplementary Figure 2), suggesting that other mechanisms regulate S100 expression.

**TABLE 1:** Correlation of S100 gain with disease stage and 1q21 gain in 168 MM patient samples.

**Variable**	**S100 gain**		**Without S100 gain**		***P***
	**No.**	**%**	**No.**	**%**	
**Disease stage**					0.0001
NDMM	4	9.8	52	40.9	
RRMM	37	90.2	75	59.1	
**1q21 gain**					<0.0001
Positive	35	85.4	43	33.9	
Negative	6	14.6	84	66.1	

*RRMM, relapsed/refractory MM patients; NDMM, new diagnosed MM patients.*

### The mRNA and Protein Expression Profile of S100 Genes in MM Samples

**FIGURE 2:**
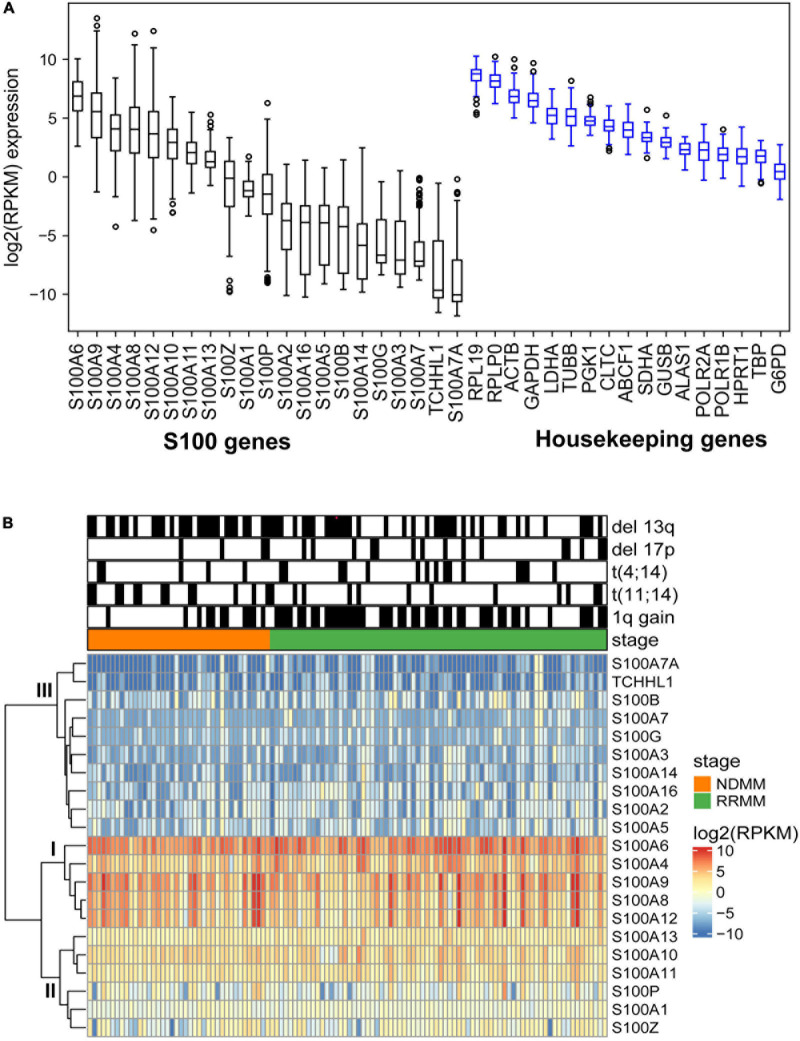
Expression profile of S100 family genes in multiple myeloma samples. **(A)** Boxplot showing the log2 (RPKM) expression of S100 and housekeeping genes from 116 MM samples. Genes are ranked based on median expression values. **(B)** Hierarchical clustering of S100 genes and patient samples using Euclidean distance matrix and complete clustering method.

To better understand S100 expression in MM, we analyzed RNA sequencing data for the expression of the S100 family members in 116 CD138+ cell samples from 95 different MM patients (Supplementary Table 4). Based on mRNA abundance in all samples, the most highly expressed S100 genes were *S100A4*, *S100A6*, *S100A9*, *S100A8*, and *S100A12* with a median log2 (RPKM) range of 6.85 (*S100A6*) to 3.67 (*S100A12*). The least abundantly expressed S100 genes were *S100A7A*, *S100A7*, *S100A14*, *S100A3*, *S100G*, *S100B*, *S100A16*, *S100A2*, and *S100A5*, with a median log2 (RPKM) range of −3.76 (*S100A2*) to −10.06 (*S1007A*) (Figure 2A). We further verified that S100 expression profiles are comparable between the CoMMpass dataset (*n* = 892) and our FIMM dataset, with a correlation coefficient of 0.70 (Supplementary Figures 3A,B). In the FIMM dataset, S100 genes clustered into three subgroups based on expression, with group I displaying the highest, group II intermediate, and group III the lowest level of expression (Figure 2B).

The levels of S100 protein were determined by mass spectrometry-based proteomics analysis of CD138+ cell samples from 35 MM patients (Supplementary Table 5). Eight S100 proteins (S100A4, S100A6, S100A8, S100A9, S100A10, S100A11, S100A12, and S100P) were identified (Supplementary Figure S3C). With the exception of S100P, all detected S100 proteins showed good correlation with gene expression level (Supplementary Figure S3D).

### Association of S100 Gene Expression With Disease Status and Patient Prognosis

Next, we investigated potential associations between S100 gene expression and clinical features (disease status, ISS stage, patient age, and gender), and with cytogenetic alterations in addition to 1q21+ in 116 MM patient samples (Supplementary Figure 4). S100 genes with an overall expression of <1 RPKM were not included in the analysis. *S100A4* and *S100A13* were more highly expressed in RRMM *versus* NDMM patient samples. *S100A6* and *S100A11* were more highly expressed in ISS stage 3 compared with ISS stage 2. Elevated expression of *S100* genes were observed to associate with certain cytogenetic makers, including *S100A6* and *S100A13* with 1q21 gain, *S100A10* with del13q, *S100A4, and S100A6* with t (4:14). In 54 paired samples from the CoMMpass dataset, *S100A4*, *S00A8*, *S100A9*, and *S100A12* showed significant expression in first relapse samples compared with the corresponding diagnostic samples (Supplementary Figure 5).

**FIGURE 3:**
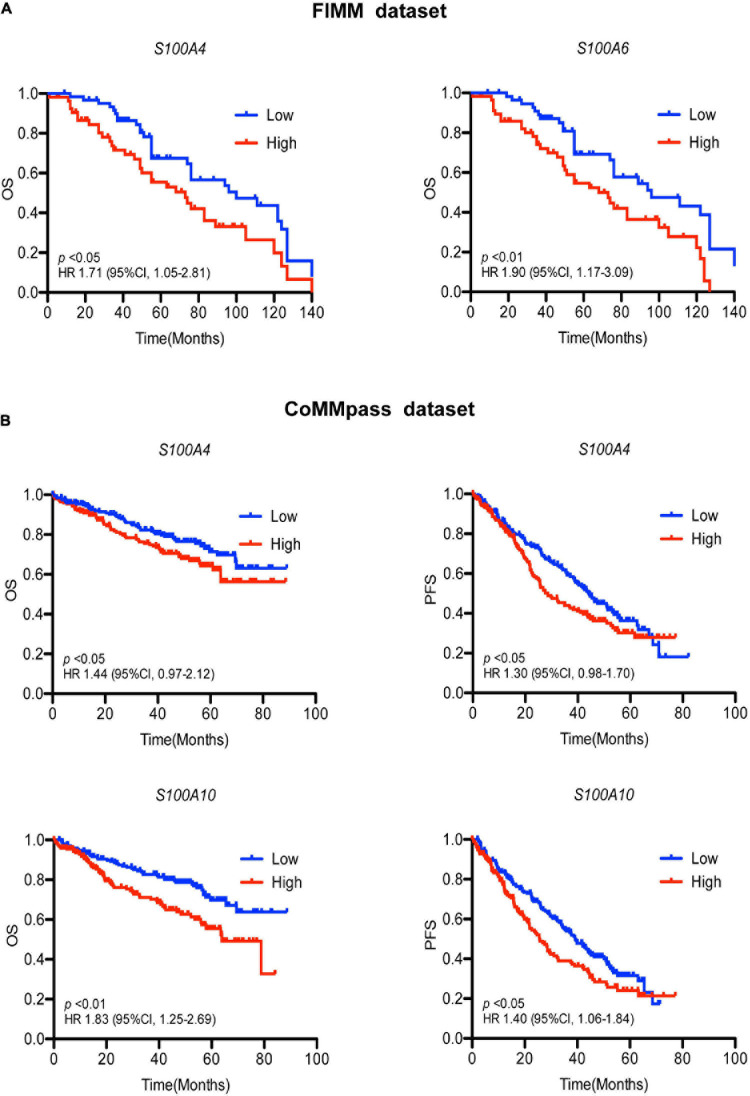
Association of S100 gene expression with patient prognosis. **(A)** High expression of *S100A4* and *S100A6* associate with poor prognosis in the FIMM dataset consisting of 116 MM samples. Low and high are defined as expression below and above the median value. **(B)** Higher expression of *S100A4* and *S100A10* are associated with shorter OS and PFS in the CoMMpass dataset including 800 MM samples. Patients were divided into groups with expression levels above the 75% percentile (high expression) and below 25% percentile (low expression). OS, overall survival; PFS, progression free survival; HR, hazard ratio; CI, confidence interval; FIMM, Institute for Molecular Medicine Finland.

To determine the effects of S100 gene expression on patient survival we performed Kaplan-Meier analysis. In the FIMM dataset, MM patients exhibiting high expression of *S100A4* and *S100A6* were more likely to have a poor prognosis (Figure 3A). The median overall survival for high vs. low expression samples was 73 vs. 100 months for *S100A4* [hazard ratio (HR) 1.71, 95% confidence limit (CL) 1.05–2.81; *p* < 0.05], and 68 vs. 96 months for *S100A6* (HR 1.90, 95% CL 1.17–3.09; *p* < 0.01). In a multivariate analysis of variables associated with OS, high *S100A4* expression retained its significance after adjusting for multiple covariates, including age, gender, cytogenetic abnormality and other S100 genes (Table 2). The association of high *S100A4* expression with poor prognosis was validated with the CoMMpass dataset (Figure 3B). In addition, high expression of *S100A10* was linked to poor survival in the CoMMpass dataset (Figure 3B), but this was not observed in the smaller FIMM cohort.

**TABLE 2:** Multivariate analysis of overall survival in the FIMM dataset (*n* = 116).

**Variable**	HR*	95% CI*	***P***
Age, years	0.94	0.90–0.98	0.002
Male	1.53	0.79–2.96	*NS*
**Cytogenetic**			
*t*(11,14)	0.34	0.13–0.90	<0.05
*t*(4,14)	1.21	0.61–2.38	*NS*
del(17p)	1.24	0.59–2.60	*NS*
del(13q)/−13	1.0	0.56–1.78	*NS*
1q gain	0.93	0.47–1.84	*NS*
**S100 gene**			
S100A4	1.40	1.10–1.77	0.006
S100A8	0.85	0.56–1.30	*NS*
S100A6	0.97	0.72–1.31	*NS*
S100A9	0.98	0.65–1.48	*NS*
S100A10	0.92	0.75–1.12	*NS*
S100A11	1.12	0.86–1.47	*NS*
S100A12	1.09	0.78–1.51	*NS*
S100A13	1.06	0.79–1.42	*NS*

*HR and 95% CIs were derived from a multivariable Cox proportional hazards regression model. All the variables in the table were included in the same model simultaneously.

### Correlation of S100 Expression With Drug Sensitivity in MM Patient Samples

**FIGURE 4:**
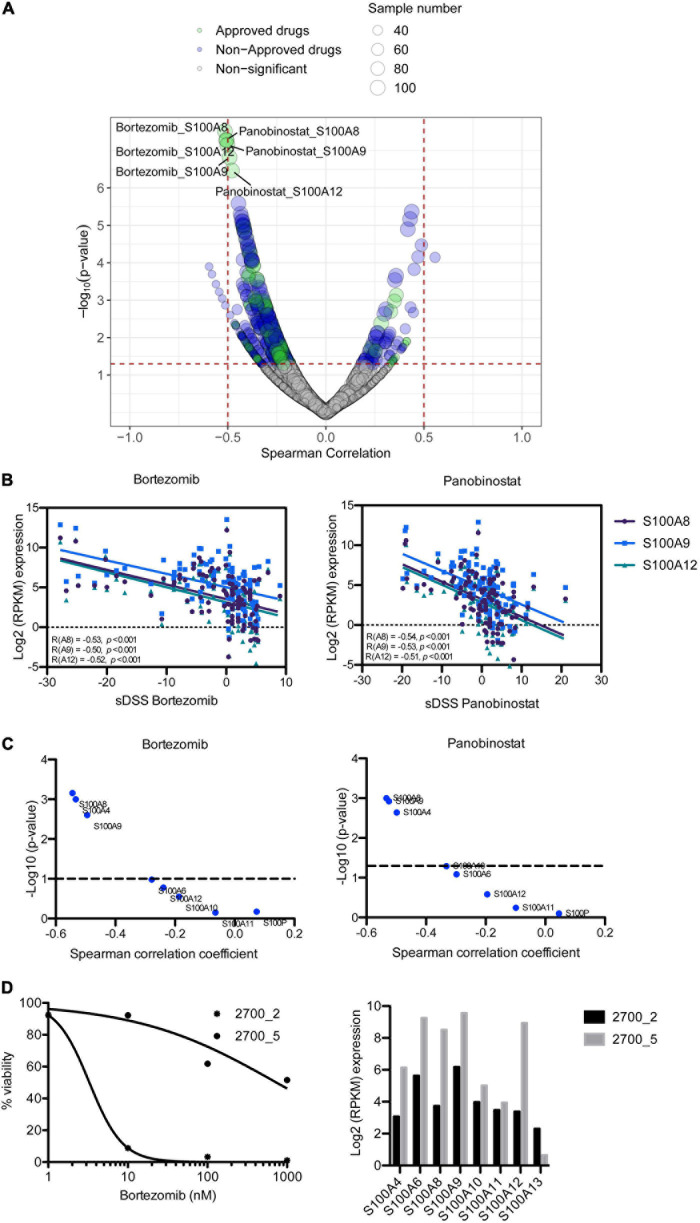
Correlation of S100 expression with *ex vivo* drug response of MM patient samples. **(A)** Representation of correlating drug sensitivity with *S100* gene expression. Dot size indicates the number of tested MM patient samples, whereas dot color indicates approval status of the drugs. **(B)** High expression of S*100A8*, *S100A9*, and *S100A12* has a negative correlation with bortezomib sensitivity. **(C)** *p*-value distribution of S100 protein associated with the sensitivity to bortezomib and panobinostat. **(D)** Bortezomib sensitivity and expression of *S100A8*, *S100A9*, and *S100A12* in serial samples from patient 2700, who had complete remission in first line (2700_2) and no response at relapse (2700_5) with bortezomib-dexamethasone-lenalidomide treatment.

To assess the impact of S100 gene expression on drug response, we correlated gene expression with *ex vivo* drug sensitivity data for 308 drugs in 106 MM patient samples. Surprisingly, we found that higher expression of *S100A8*, *S100A9*, and *S100A12* inversely correlated with sensitivity to two approved MM drugs, the PI bortezomib and HDAC inhibitor panobinostat, with a Spearman’s correlation coefficient around 0.5 and false discovery rate (FDR) < 0.05 (Figures 4A,B and Supplementary Table 6). Expression of these *S100* genes significantly negatively correlated with the sensitivity to other approved PIs including carfilzomib and ixazomib (Supplementary Table 6). The inverse correlation between S100 gene expression and drug sensitivity extended to other inhibitors including those targeting PI3K, IGF1R, PKC and PI3K/mTOR (Supplementary Table 6). However, considering drugs approved for MM, we focused on PIs and panobinostat for further study. A comparison of proteomic and drug sensitivity data from 35 MM patients also showed that the level of S100A8 and S100A9 proteins negatively correlated with bortezomib and panobinostat sensitivity (Figure 4C and Supplementary Table 7). Moreover, when accounting for 100 genes located on chromosome 1q21, the associations of the genes in this region with PI sensitivity were analyzed. The data showed S100 members were on the top list of PI-correlated genes, in comparison to other genes with 1q21 region (Supplementary Table 8), highlighting the important role of S100 genes in PI response.

Using a set of two samples taken from the same patient at diagnosis and at relapse when the patient had become resistant to bortezomib-based treatment, we analyzed for changes in the level of *S100A8*, *S100A9*, and *S100A12* expression. In accordance with the clinical response, our *ex vivo* analysis showed exceptional sensitivity to bortezomib in pre-treatment sample (2700_2), but resistant in the sample taken at relapse (2700_5) (Figure 4D). Furthermore, *S100A8, S100A9*, and *S100A12* were more highly expressed in the bortezomib resistant sample than in the pre-treatment sample (Figure 4D).

### Clinical Relevance of S100A4 Overexpression for Carfilzomib Resistance

**FIGURE 5:**
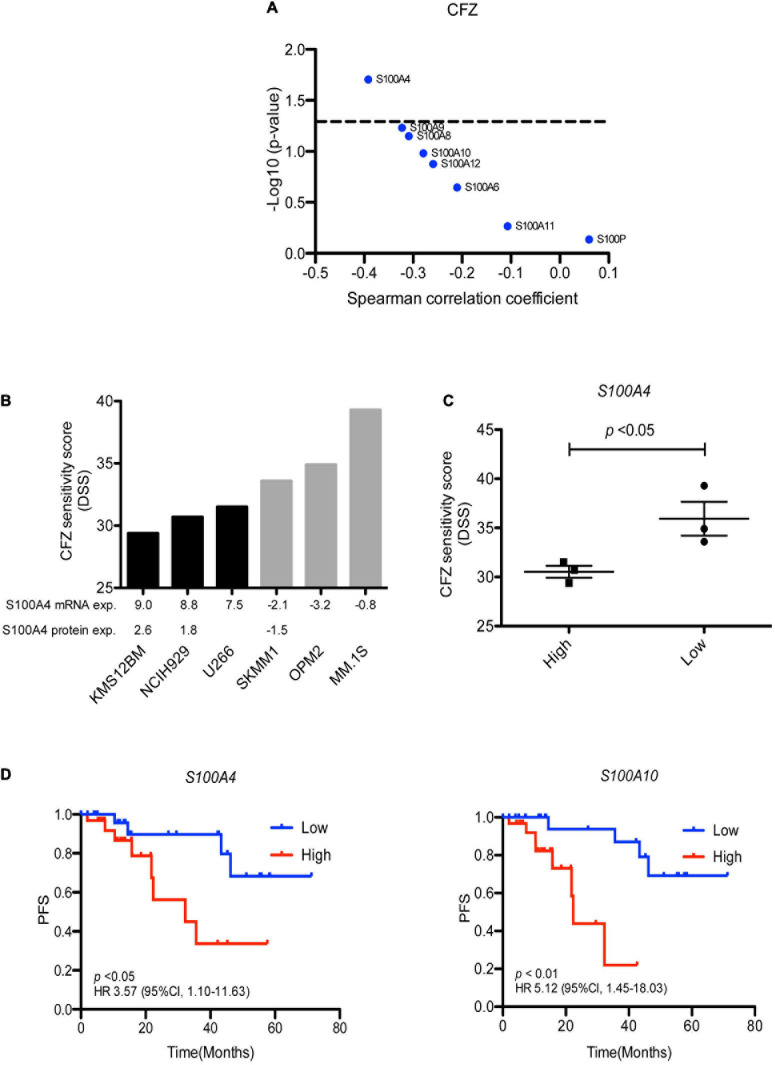
S100A4 overexpression linked to carfilzomib resistance. **(A)** Correlation of S100 protein expression with *ex vivo* carfilzomib sensitivity. **(B)** CFZ score in the MM cell lines having high and low–level expression of S100A4. Gene and protein expression presented as log_2_ FPKM value and log_2_ LFQ intensity, respectively. **(C)** Statically analysis of CFZ DSS value in the MM cell lines expressing high vs. low expression. **(D)** High expression of *S100A4* and *S100A10* correlated with shorter progression free survival in 63 MM patients, who received first-line carfilzomib-based therapy from the CoMMpass dataset. Low and high are defined as expression below and above the median value. CFZ, carfilzomib; DSS, drug sensitivity score; PFS, progression free survival.

Analysis of proteomic data revealed that high expression of S100A4 protein significantly correlated to *ex vivo* resistance to carfilzomib in 35 MM patients (Figure 5A). To validate this finding, we collected *S100A4* expression data of several MM cell lines from the Keats Lab^7^, and performed drug sensitivity testing of the same cell lines with different PIs. The analysis showed response to carfilzomib was much better in *S100A4* low cell lines (SK-MM-1, OPM-2, and MM.1S) vs. *S100A4* high expressing cell lines (KMS-12-BM, NCI-H929, and U-266) (Figures 5B,C). In addition, combining proteomic data from the DepMap portal^8^ and our drug sensitivity data, we found that the cell lines KMS-12-BM and NCI-H929 which have high levels of S100A4 protein were more resistant to carfilzomib compared to the OPM-2 cell line which had lower levels of S100 protein expression (Figure 5B). Furthermore, to explore the clinical relevance of this finding, we assessed for association of *S100A4* gene expression and survival of MM patients (*n* = 63) who received first line carfilzomib-based therapy in the CoMMpass dataset. Survival analysis revealed high expression of *S100A4* and *S100A10* linked to shorter progression free survival (PFS), with median PFS: 14.6 vs. 25.4 months for *S100A4* and 11.5 vs. 28 months for *S100A10* (Figure 5D).

## Discussion

Chromosome 1q21 gain, a common genetic abnormality in MM, is associated with poor prognosis in MM (An et al., 2014). Although some genes within this region have been shown to impact MM disease progression (Shaughnessy, 2005), the molecular mechanisms resulting in 1q21 gain and whether it is a cause or a consequence of the disease remain elusive. Seventeen S100 genes are in the chromosome 1q21 region, all of which could potentially contribute to cancer progression (Bresnick et al., 2015). In this study, we systematically studied the roles of S100 family members in MM at the copy number, transcriptional and protein levels using data collected from individual patients as well as paired data from the same patients.

We studied a cohort of 168 MM patient samples and found that 24% displayed copy number gains of S100 genes, including S100A1, S100A2, S100A3, S100A4, S100A5, S100A6, S100A7, S100A7A, S100A8, S100A9, S100A10, S100A11, S100A12, S100A13, S100A14, S100A16, and TCHLL. Survival analysis also revealed the gains of these S100 genes associated with poor patient prognosis in our FIMM dataset and the MMRF CoMMpass dataset. It is widely accepted that copy number abnormalities confer a selective advantage for tumor development and metastasis (Weaver and Cleveland, 2007; Bolli et al., 2018). Our results indicated prognostic roles of S100 copy number changes in MM patients. However, for most S100 genes, copy number changes did not significantly correlate with mRNA expression. The mRNA expression profiles showed high expression of S100A4, S100A6, S100A8, S100A9, and S100A12, and low expression of S100A7A, S100A7, S100A14, S100A3, S100A16, S100A2, and S100A5, although all are in the 1q21 region and had similar copy number gains in 116 MM patient samples. Numerous regulatory mechanisms can control and disrupt mRNA transcription, such as transcription factors, microRNAs and epigenetic modifiers, besides CNV. For example, DNA hypomethylation resulted in reduced S100A6 and S100A10 expression in medulloblastoma while somatic methylation controlled S100A2 and S100A4 expression in the normal cerebellum, showing tissue specific regulation (Lindsey et al., 2007). Diminished S100A2 expression was shown to correlate with increased methylation in small cell lung cancer cells and metastatic head and neck cancer (Leśniak, 2011). Downregulation of miR-520c induced S100A4 expression in colorectal cancer (Giridhar et al., 2017). Furthermore, S100 expression can be induced by certain growth factors, cytokines, toll-like receptor (TLR) ligands, and intracellular signaling cascades. For example, the pro-inflammatory cytokine IL-10 can upregulate S100A8 expression (Endoh et al., 2009) and integrin signals via Src kinase lead to increased S100A4 expression (Kim et al., 2009). We observed high correlation between mRNA and protein expression levels, suggesting that mRNA expression levels of S100 genes are reflective of protein level expression in MM.

In the second part of this study, we explored the clinical relevance of S100 expression in MM. *S100A4* and *S100A13* were significantly more highly expressed in RRMM vs. NDMM patients. In serial samples from the CoMMpass data, *S100A4* also showed significant expression in first relapse samples vs. the corresponding diagnostic samples. Survival analysis showed elevated expression of *S100A4* could predict poor survival of MM patients in our FIMM dataset and the MMRF CoMMpass dataset. *S100A4* remained an independent predictor of inferior survival after adjusting for multiple variables, including the high-risk cytogenetic abnormalities. *S100A4* (also known as a metastasin-1), a metastasis -associated protein and an epithelial to mesenchymal transition (EMT) marker, promotes cell survival and proliferation, angiogenesis, invasion and metastasis, and tumor-promoting inflammation and has been implicated in cancer progression and metastasis (Helfman et al., 2005; Sherbet, 2009). *S100A4* has been reported to be expressed in the nucleus, cytoplasm, and extracellular space of many cells. Once secreted by the cell, extracellular S100A4 can activate NF-κB and participate in paracrine and/or autocrine signaling through binding to their receptor TLR4 and RAGE as well as other cell surface receptors (Donato et al., 2013). It is possible the effects of *S100A4* on MM progression and patient survival by its extracellular and intercellular functions. *S100A13* acts as an angiogenic marker for melanoma (Massi et al., 2010; Azimi et al., 2014) and has been involved in invasiveness of lung cancer cell lines (Miao et al., 2018). The functions of *S100A13* in MM remain unclear and need further investigations.

Lastly, we integrated *S100* gene expression and *ex vivo* drug sensitivity data, including 308 approved and investigational drugs, to determine whether the expression of *S100* genes correlate with drug response, especially established myeloma drugs. The associations of *S100* gene expression with our *ex vivo* drug sensitivity data revealed *S100A8*, *S100A9*, and *S100A12* expression inversely correlated with bortezomib, carfilzomib and panobinostat sensitivity, all of which have been approved for the treatment of MM. Analysis of S100 protein levels confirmed a similar pattern with mRNA levels. In a paired set of diagnosis-relapse samples from the same patient, these genes were more highly expressed after the patient became resistant to bortezomib-based treatment. In line with our findings, a previous study showed inhibition of S100A9 results in decreased viability and proliferation of MM cells and overcomes resistance to bortezomib (De Veirman et al., 2017). S100A8 and S100A9 exist as homodimers and heterodimers. S100A8/S100A9 (also called calprotectin) has both intercellular and extracellular functions. Upregulation of *S100A8* and *S100A9* occurs in various human cancer types and may participate in tumor growth, metastasis, angiogenesis and immune evasion (Ichikawa et al., 2011; Grebhardt et al., 2014; Goh et al., 2017; Mondet et al., 2021). Elevated expression of *S100A8*/*S100A9* caused glucocorticoid resistance in MLL rearranged infant acute lymphoid leukemia (Spijkers-Hagelstein et al., 2012) and negatively associated with BCL2 inhibitor venetoclax in AML (Karjalainen et al., 2019). Our data suggest *S100A8* and *S100A9* expression may predict PI response and targeting S100A8 or S100A9 may have therapeutic value. In addition, a negative association between S100A4 protein expression and carfilzomib sensitivity was observed in our dataset and in MM cell lines we found that higher S100A4 expression also linked to carfilzomib resistance. From the CoMMpass dataset higher *S100A4* gene expression significantly correlated with lower PFS for MM patients who received carfilzomib-based therapy, suggesting S100A4 maybe a therapeutic target for MM patients. We acknowledge, however, that this finding requires further exploration. Previous studies showed knockdown of the *S100A4* gene could alter cell metabolism and promote invasion of lung cancer (Liu et al., 2019), restrict metastasis formation in colorectal cancer (Dahlmann et al., 2012), reduce the proliferative and invasive capacities of highly invasive prostate cancer cells (Saleem et al., 2006), and resulted in cell death in leukemia cells (Alanazi et al., 2020). Overexpression of *S100A4* conferred human cancer cell lines resistant to the chemotherapeutic drug methotrexate (Mencía et al., 2010) and suppression of the *S100A4* gene increased sensitivity of pancreatic cancer cells to gemcitabine treatment (Mahon et al., 2007). In a recent study, *S100A4* showed significant upregulation in MM non-responding vs. responding patients treated with KARA-KRD (Cohen et al., 2021). These studies indicate the potential role of S100A4 gene in MM progression and drug response, and suppression of *S100A4* gene may contribute to a novel therapeutic approach for MM treatment.

Our study has limitations. Notably, most patients received a PI combined with other therapeutic agents, making it is difficult to make a direct connection between S100 expression and clinical PI response. However, our drug sensitivity assays testing individual PIs with primary MM patient cells and with MM cell lines showed a clear association between S100 expression and PI sensitivity. Nevertheless, gain- and loss- of function experiments are needed to study whether the S100 genes are a causal factor in MM progression or PI and panobinostat resistance.

## Conclusion

In conclusion, gains of S100 genes at 1q21 are frequent in MM and associate with poor prognosis. High expression of S100 members in MM such as *S100A4*, *S100A6*, *S100A8*, *S100A9*, and *S100A12*, indicate a possible role in disease development, while higher expression *S100A4* and *S100A13* in RRMM vs. NDMM suggest a role in disease progression. Furthermore, higher expression of *S100A4*, *S100A8*, *S100A9*, and *S100A12* correlated with reduced *ex vivo* sensitivity to PIs and panobinostat. The results were corroborated clinically where higher expression of *S100A4* was significantly linked to shorter PFS in patients given carfilzomib-based therapy. Overall, these data indicate that S100 family members have predictive value in MM, may potentially adapt MM cells to PI-based treatment, and could be potential therapeutic targets.

## Data Availability Statement

The datasets presented in this study can be found in online repositories. The names of the repository/repositories and accession number(s) can be found in the article/Supplementary Material.

## Ethics Statement

The studies involving human participants were reviewed and approved by an Ethical Committee of the Helsinki University Hospital Comprehensive Cancer Center (permit numbers 303/13/03/01/2011 and 239/13/03/00/2010) and conducted following the guidelines of the Declaration of Helsinki. The patients/participants provided their written informed consent to participate in this study.

## Author Contributions

ML and CH designed the study. YW contributed substantially to data analysis and visualization. ML wrote the manuscript. CH participated in the writing and editing of the manuscript, and supervised the study. All authors contributed to data acquisition, analysis, or interpretation, and reviewed and approved the final version of the manuscript.

## Conflict of Interest

CH has received funding from BMS/Celgene, Kronos Bio, Novartis, Oncopeptides, Orion Pharma, and the IMI2 projects HARMONY and HARMONY PLUS. RS has received research funding from Amgen, BMS, Celgene and Takeda, and honoraria from Amgen, Celgene, Janssen-Cilag, Takeda, and Sanofi. JL and PA have received personal fees from Amgen, Bristol-Myers Squibb, Celgene, Janssen, Sanofi, and Takeda. The remaining authors declare that the research was conducted in the absence of any commercial or financial relationships that could be construed as a potential conflict of interest.

## Publisher’s Note

All claims expressed in this article are solely those of the authors and do not necessarily represent those of their affiliated organizations, or those of the publisher, the editors and the reviewers. Any product that may be evaluated in this article, or claim that may be made by its manufacturer, is not guaranteed or endorsed by the publisher.

## Footnotes

1
https://osf.io

2
https://github.com/chrisamiller/copycat

3
https://www.maxquant.org

4
https://www.maxquant.org/perseus/

5
https://research.themmrf.org

6
www.themmrf.org

7
https://www.keatslab.org/data-repository

8
https://depmap.org/portal/interactive/
