# Supplementary material for: S100 Calcium Binding Protein Family Members Associate With Poor Patient Outcome and Response to Proteasome Inhibition in Multiple Myeloma
Source: Front Cell Dev Biol. 2021 Aug 16;9:723016. doi: 10.3389/fcell.2021.723016 (PMC8415228; doi:10.3389/fcell.2021.723016)
Supplement: Supplementary file 1 [file Data_Sheet_1.pdf]

Supplementary Material

TABLE OF CONTNETS

Supplementary Figures

Figure S1. Prevalence and prognostic significance of *S100* copy number change in the CoMMpass dataset (n=1044).

Figure S2. Correlation of *S100* copy number variants with mRNA expression from the FIMM dataset.

Figure S3. *S100* gene family genes showed similar expression in both the CoMMpass and FIMM datasets.

Figure S4. Correlation of *S100* genes with myeloma patient stage, ISS stage, cytogenetics (1q21 gain, del(13q), del(17p), t(4:14) and t(11:14)), gender and age in the FIMM dataset.

Figure S5. S100 gene expression in the 54-paired diagnostic and 1st relapsed multiple myeloma patients from the CoMMpass data.

Supplementary Tables

Table S1. Clinical characteristics of the patients in the current study.

Table S2. S100 gene copy number variation scores from MM patient samples in the FIMM dataset (n=168).

Table S3 Statistical overview of *S100* copy number variations with patient outcome in the FIMM dataset (n=168).

Table S4. Log2 (RPKM) expression values for 21 S100 genes and 17 housekeeping genes in MM patient samples in the FIMM dataset (n = 116)**.**

Table S5. LC-MS/MS-based proteomics label free quantitation intensity values for eight S100 proteins in CD138+ cells isolated from MM patient samples in the FIMM dataset (n = 35).

Table S6. Correlation of high mRNA expression of S100 members with response to 308 drugs (sDSS).

Table S7. Correlation of S100 protein expression with response to Bortezomib, Carfizomib and Panobinostat (n = 35).

Table S8. Significantly correlated genes within the 1q21 region involved in the response to proteasome inhibitors.

Supplementary Figures

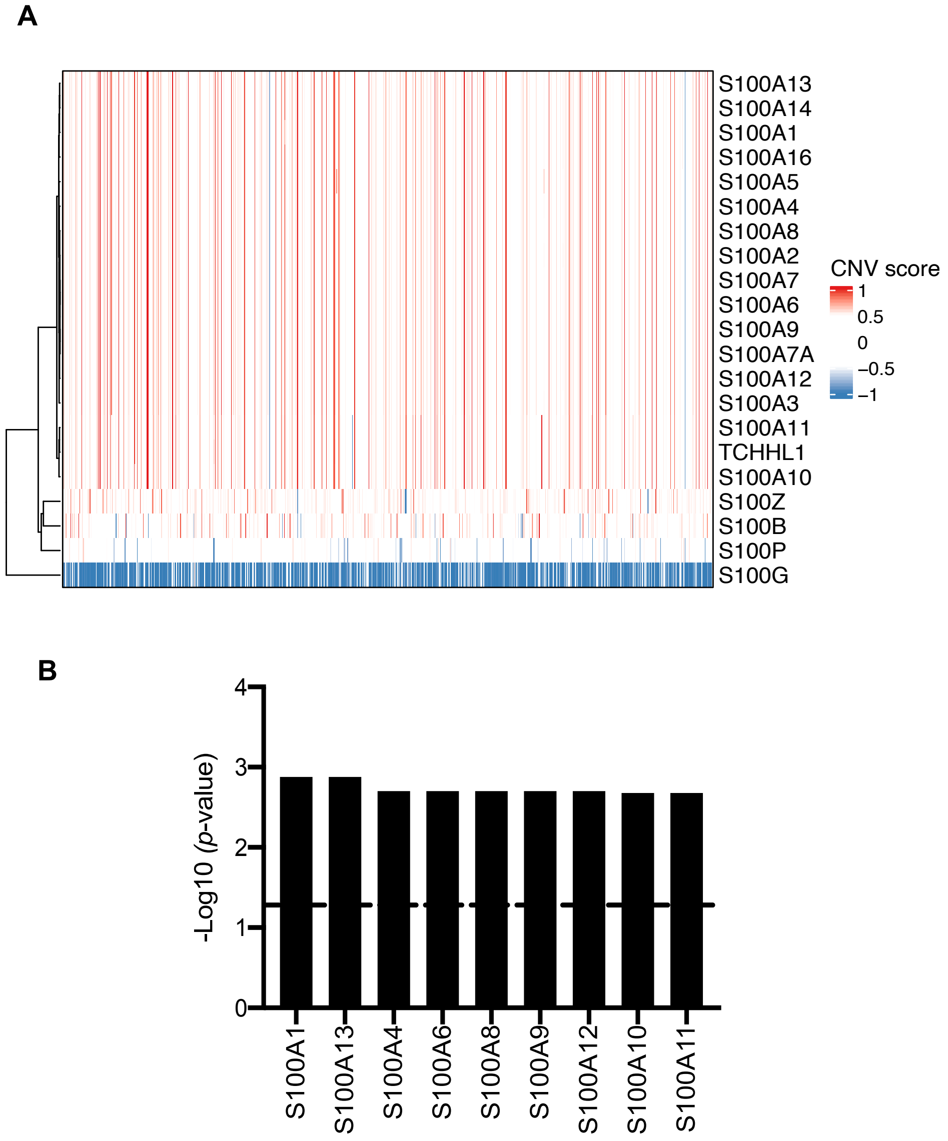

Figure S1. Prevalence and prognostic significance of *S100* copy number change in the CoMMpass dataset (n=1044). (A) Copy number alterations of *S100* genes from the CoMMpass dataset. (B) Log-rank test p-values in overall survival analysis related to copy number gain of *S100* genes from the CoMMpass dataset. CNV: copy number variation; CNV score > 0.5 indicated a gain event, while < -0.6 was a deletion event.

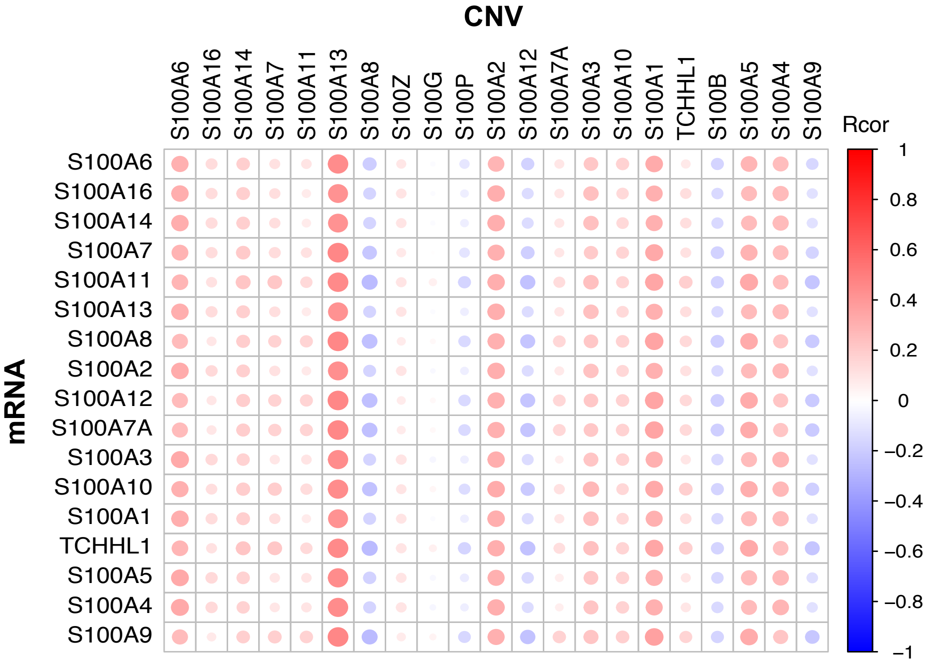

Figure S2. Correlation of *S100* copy number variants with mRNA expression from the FIMM dataset. FIMM: Institute for Molecular Medicine Finland; CNV: copy number variation. Rcor: correlation coefficient. Red and blue represented positive and negative relationship, respectively. Dot size indicated the strength of a correlation.

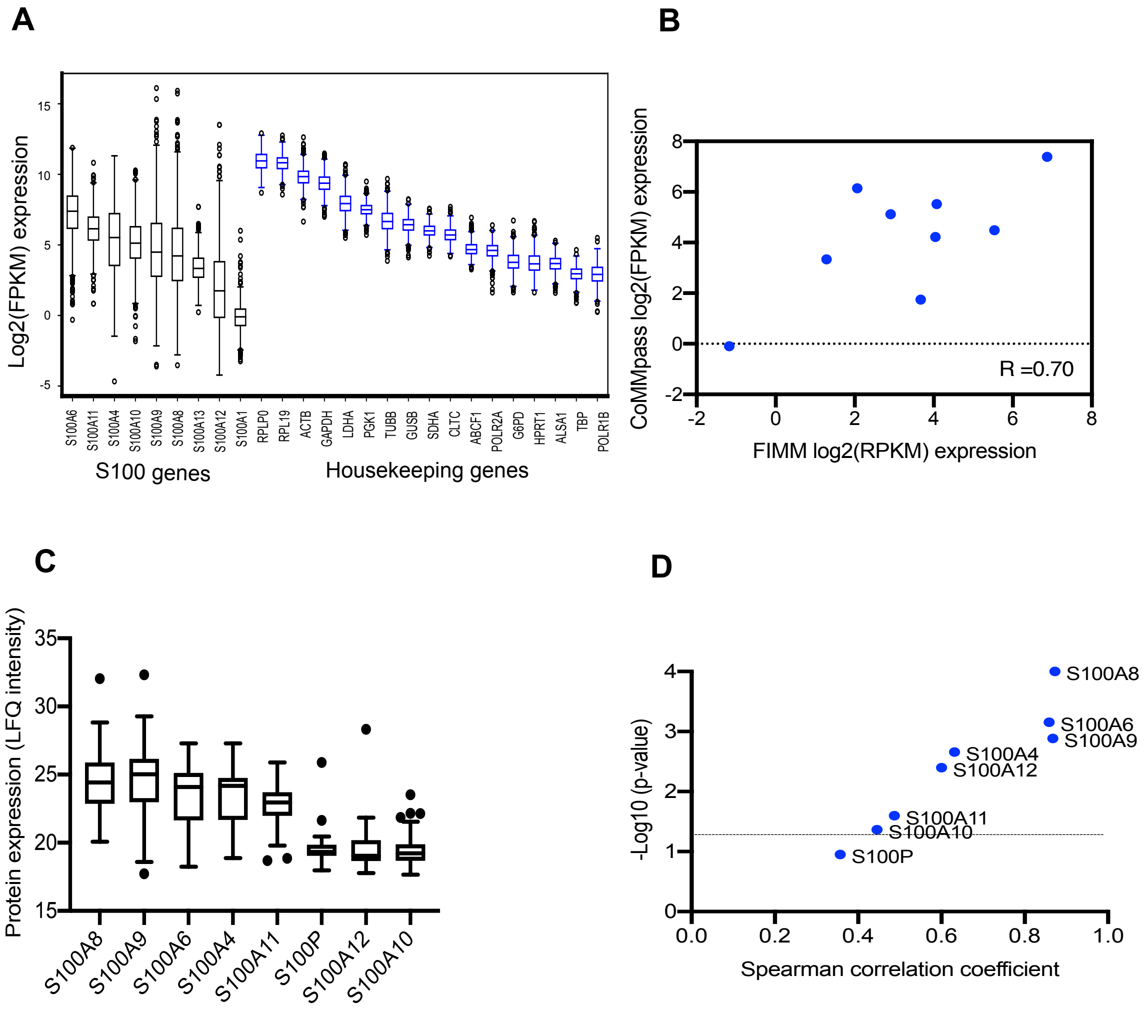

Figure S3. ***S100* gene family genes showed similar expression in both the CoMMpass and FIMM datasets.** (A) Boxplots showing *S100* genes expression in the CoMMpass dataset (n=892). (B) Correlation of median *S100* gene expression between the FIMM dataset and the CoMMpass dataset. (C) S100 protein expression profile in 35 MM samples, detected by LC-MS/MS. (D) Correlation of protein and gene expression levels for the eight *S100* genes. FPKM: fragments per kilobase of transcript per million mapped reads; LC-MS/MS: liquid chromatography-tandem mass spectrometry; LFQ: label-free quantification.

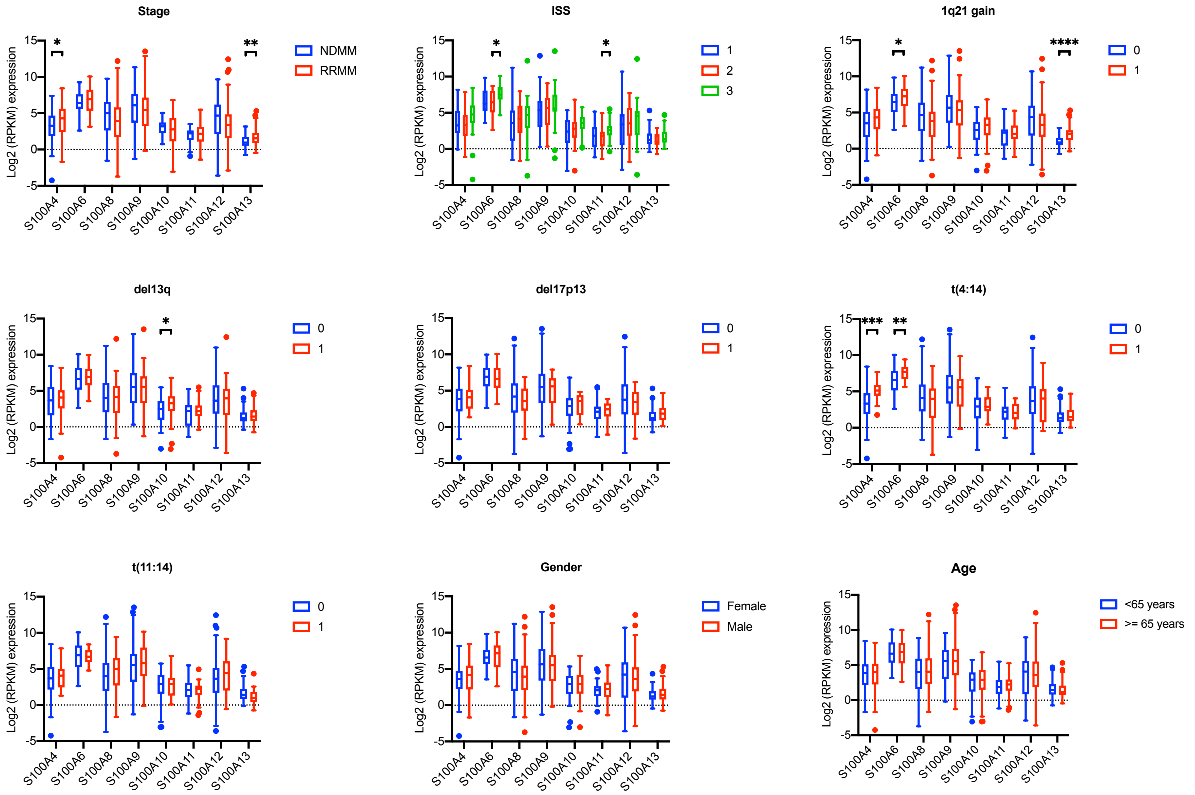

Figure S4. Correlation of *S100* genes with myeloma patient stage, ISS stage, cytogenetics (1q21 gain, del(13q), del(17p), t(4:14) and t(11:14)), gender and age in the FIMM dataset. Statistical significance was denoted as * $p<0.05$, ** $p< 0.01$, *** $p<0.001$, **** $p<0.0001$. RPKM: reads per kilobase of transcript per million mapped reads.

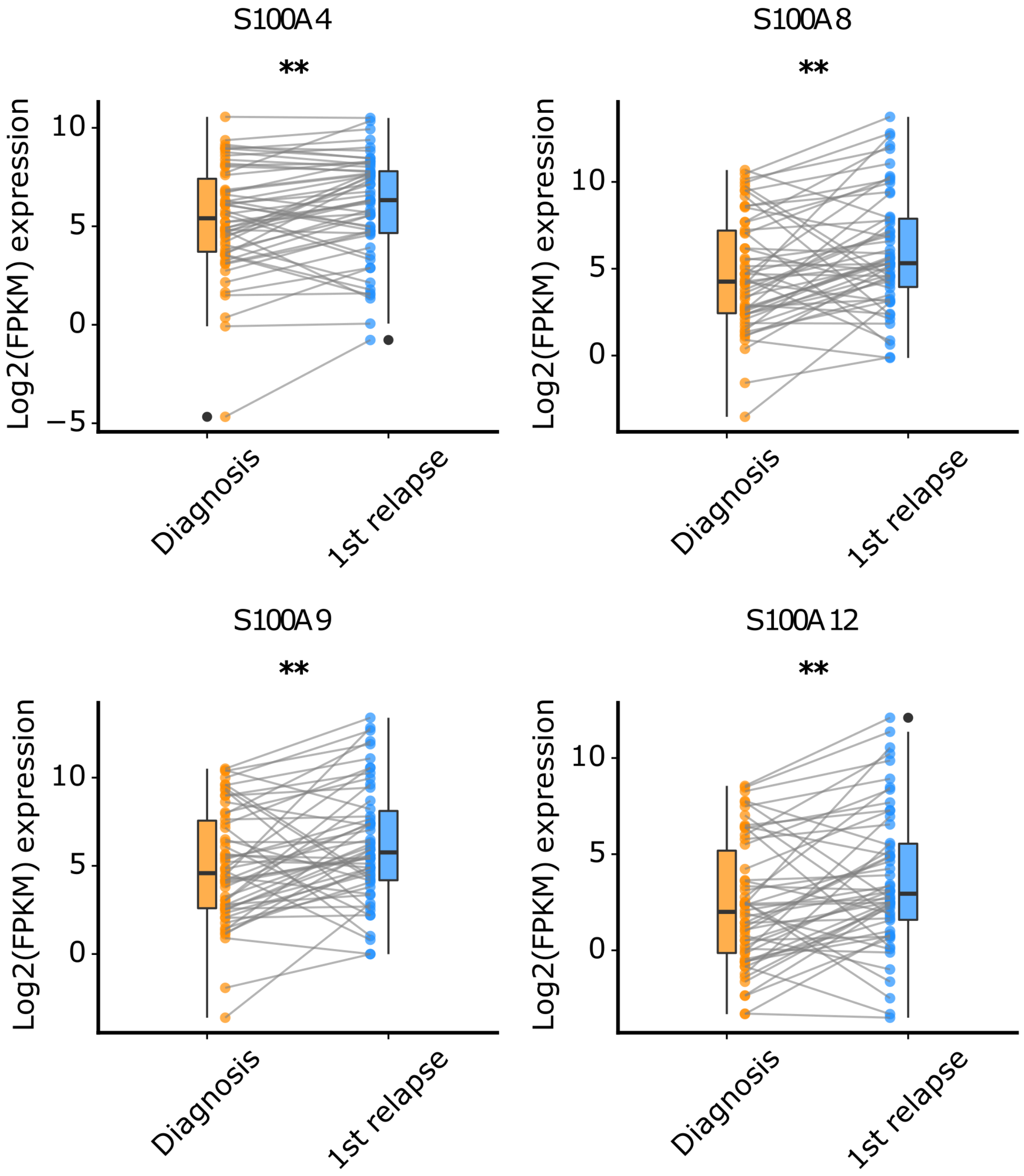

Figure S5. S100 gene expression in the 54-paired diagnostic and 1st relapsed multiple myeloma patients from the CoMMpass data. Differences between the two stages were statically analysed by paired-t-tests. Statistical significance was denoted as * p<0.05, ** p< 0.01, *** p<0.001, **** p<0.0001. FPKM: fragments per kilobase of transcript per million mapped reads.

Supplementary Tables are uploaded separately as an excel file.
